# Phenolic Compounds Profile and Antioxidant Capacity of Pitahaya Fruit Peel from Two Red-Skinned Species (*Hylocereus polyrhizus* and *Hylocereus undatus*)

**DOI:** 10.3390/foods10061183

**Published:** 2021-05-25

**Authors:** Wanpei Tang, Wu Li, Yuzhe Yang, Xue Lin, Lu Wang, Congfa Li, Ruili Yang

**Affiliations:** 1 Key Laboratory of Food Nutrition and Functional Food of Hainan Province, College of Food Science and Engineering, Hainan University, Haikou 570228, China; 18083200210009@hainanu.edu.cn (W.T.); leewuu@163.com (W.L.); lwang@hainanu.edu.cn (L.W.); congfa@vip.163.com (C.L.); 2 College of Food Science, South China Agricultural University, Guangzhou 510642, China; yz3794847@163.com

**Keywords:** bound phenolics, pitahaya peel, antioxidant capacity, hydrolysis

## Abstract

Pitahaya peel is a good source of bioactive polyphenols. However, the bound phenolics and their antioxidant activity remain unclear. The bound phenolics of pitahaya peel from two red-skinned species with red pulp (RP) and white pulp (WP) were released with different methods (acid, base, and composite enzymes hydrolysis). The results revealed that base hydrolysis was the most efficient method for releasing the bound phenolics from RP (11.6 mg GAE/g DW) and WP (10.5 mg GAE/g DW), which was 13.04-fold and 8.18-fold for RP and 75.07-fold and 10.94-fold for WP compared with acid hydrolysis and enzymatic hydrolysis, respectively. A total of 37 phenolic compounds were identified by UPLC-TOF/MS with most chlorogenic acid, caffeic acid, ferulic acid and *p*-coumaric acid in RP, whereas chlorogenic acid, caffeic acid, ferulic acid, rutin and isoquercitrin were the main compounds in WP. Regardless of the hydrolysis method, the extracts having the highest phenolic content showed the strongest antioxidant activities. The work shows that hydrolysis methods have a significant effect on the release of phenolics, and the contents of major characteristic bound phenolic compounds are related to the ecological type of pitahaya.

## 1. Introduction

*Hylocereus* is a native American cactus of varied habits and widely distributed from the Florida coast to Brazil. The fruit is known under several commercial and native names, but “pitahaya” or “pitaya” prevails all around [1]. Among them, red pitahaya (*Hylocereus* spp.), fruits with a red skin and red (*Hylocereus polyrhizus*) or white (*Hylocereus undatus*) pulp are the most common commercial species [2]. In recent years, with the improvement of people’s living standards and health consciousness, fruit products for high nutrients have been favored by consumers. Pitahaya has attracted attention because it is a promising source of nutrients of exerting positive effects on the consumers’ health. Pitahaya peel, accounting for approximately 33% of the whole fruit weight, is often discarded and ends up as waste in the process of industrial processing, which causes considerable environmental pollution [3].

It is a truth universally acknowledged that fruit peel is a good source of bioactive compounds, such as phenolics [4,5]. Phenolic compounds have attracted much attention because of their potential healthy effects, such as antioxidant, anticancer cell and anti-diabetes activities [6,7]. In general, natural phenolic compounds that exist in plant matrices occur mostly as free and bound forms (covalently bound to sugar moieties or cell wall structural components) [8]. Free phenolics can easily be extracted from various matrices, while bound phenolic compositions which occupy about 35–65% of the phenolic compounds in plant matrices are usually released by acidic hydrolysis, saponification (base/alkaline hydrolysis) and enzymatic reactions [9,10,11,12]. Alkaline hydrolysis is the commonly strategy to release the bound phenolics, which entails treating the sample with NaOH solution (about 1–4 M) for 15 min up to overnight. As for acidic hydrolysis, it was efficient to break glycosidic bond and then to release the bound form phenolics [13]. Currently, composite enzymatic treatment has been in the spotlight to extract bound phenolics for the low cost and environmentally friendly feature. However, due to the differences of plant materials and substrates, there are differences in the ability of these methods to release bound phenolics [14,15,16].

It has previously been observed that the total free phenol content of red pitahaya peel and white pitahaya peel were 14.82 mg/g and 15.94 mg GAE (gallic acid equivalent)/g of dried sample [17]. Currently, over 23 polyphenol compounds have been identified from flesh and peel of red pitahaya fruits (gallic acid, protocatechuic acid, vanillic acid, caffeic acid, syringic acid, *p*-coumaric acid, isorhamnetin triglycoside, quercetin-3-O-rutinoside, flavonol glycoside and so on) by HPLC-DAD and HPLC–MS–MS [3,18]. However, most previous studies on pitahaya peel have focused only on free phenolic compounds [18,19,20]. To our knowledge, the bound phenolic compounds and their contributions to total phenolic compounds and antioxidant activities remain to be elucidated.

The aim of this study was to compare the efficiency of the acid, base, and composite enzymes hydrolysis in releasing bound phenolic compounds from red-pulp and white-pulp pitahaya peel. The released phenolic compounds resulting from different extraction methods were identified and quantified using ultra performance liquid chromatography (UPLC) and UPLC-Q-Exactive Orbitrap mass spectrometry. Moreover, the antioxidant activity of phenolic fractions was also investigated.

## 2. Materials and Methods

### 2.1. Chemicals and Reagents

Epicatechin, kaempferol, quercetin, rutin, isoquercitrin, syringic acid, *p*-coumaric acid, chlorogenic acid, cryptochlorogenic acid, caffeic acid, ferulic acid and gallic acid were purchased from Adamas Reagent, Ltd. (Shanghai, China). Other standard reference materials including 4-methoxysalicylic acid, *p*-hydroxycinnamic acid, isoferulic acid, 1,3-dicaffeoylquinic acid, gentiopicrin, grosvenorine, diosmin, isorhamnetin and baicalein were obtained from Shanghai Yuanye Bio-Technology Co., Ltd. (Shanghai, China). 2,4,6-tri(pyridin-2-yl)-1,3,5-triazine (TPTZ), 1,1-diphenyl-2-picrylhydrazyl (DPPH), 2,2′-azino-bis (3-ethylbenzothiazoline-6-sulfonic acid (ABTS), 4-nitrophenyl α-D-glucopyranoside (*p*-NPG) were all obtained from Adamas Reagent, Ltd. (Shanghai, China). Acetonitrile and formic acid (99.9%, HPLC grade) were obtained from Thermo Fisher Scientific (Waltham, MA, USA). Cellulase (400 u/mg) from *Trichoderma Vride G*, hemicellulase (20,000 u/g) and pectinase (500 u/mg) from *Aspergillus niger* were all supplied from Shanghai Yuanye Bio-Technology Co., Ltd. (Shanghai, China). All other reagents and chemicals used in this study are analytical grade.

### 2.2. Plant Materials Collection and Preparation

Red-pulp and white-pulp pitahaya fruits at commercial maturity, no mildew and deterioration, were purchased from Haikou Ledong Pitahaya Planting Base (Ledong, China). Red-pulp pitahaya peel (RP) and white-pulp pitahaya peel (WP) were separated and lyophilized and powdered to a fine powder and stored at −20 °C until analysis.

### 2.3. Different Extraction Methods

#### 2.3.1. Free Phenolics by Methanol

**Figure 1:**
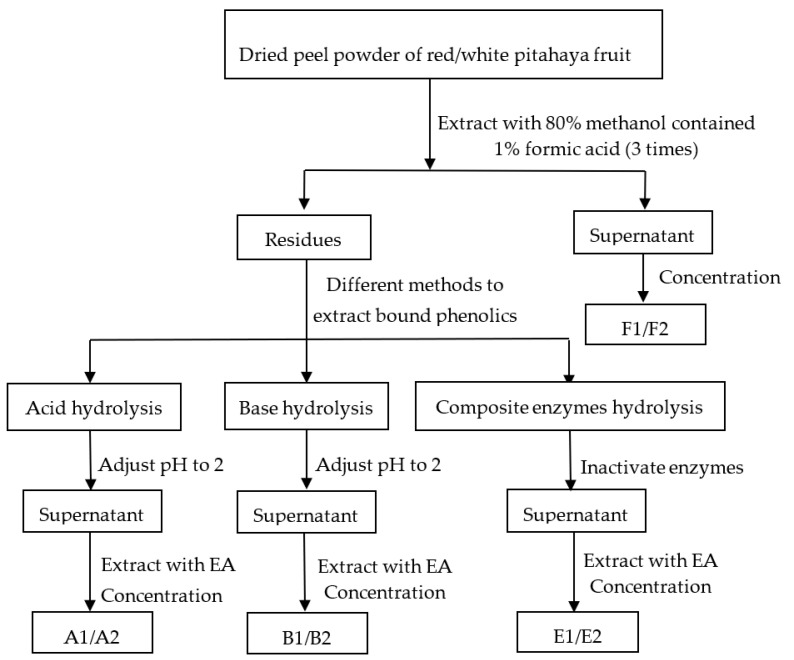
Phenolic fractions extraction procedures. EA, Ethyl acetate; F1, free phenolics of red pitahaya peel by methanol extraction; F2, free phenolics of white pitahaya peel by methanol extraction; A1, bound phenolics of red pitahaya peel by acid hydrolysis; A2, bound phenolics of white pitahaya peel by acid hydrolysis; B1, bound phenolics of red pitahaya peel by base hydrolysis; B2, bound phenolics of white pitahaya peel by base hydrolysis; E1, bound phenolics of red pitahaya peel by composite enzymes hydrolysis; E2, bound phenolics of white pitahaya peel by composite enzymes hydrolysis.

Extraction of free phenolics from the RP and WP was performed according to Li et al. [21] (Figure 1). Briefly, the dried pitahaya peel powder (0.5 g) were mixed with 15 mL of 80% methanol solution (including 1% formic acid). Ultrasonic cleaner (KQ-500DE, Kun shan, Jiangsu, China) was used to ultrasonic the mixture at 400 W power and 25 °C for 30 min. The supernatant was collected after centrifugation at 4000 r/min for 15 min. Residues were re-extracted twice as above. The supernatants were combined and concentrated with a rotary evaporator at 45 °C avoiding light, and the resulting extracts were dissolved in 70% methanol (final volume, 10 mL) for determination. This was the free phenolics of RP and WP (F1 and F2).

#### 2.3.2. Bound Phenolics by Acid Hydrolysis

The dried residue (0.5 g) generated after free phenolics extraction was hydrolyzed with 15 mL of HCl (3 M) filled with nitrogen. The mixture was kept in water bath at 85 °C for 60 min and then adjusted the pH to 2 using NaOH solution (10 M), centrifuged at 10,000× *g* for 5 min. The supernatant was extracted with 20 mL of ethyl acetate (EA) four times. All of the supernatants were concentrated with a rotary evaporator at 45 °C avoiding light, and used 70% methanol to constant volume of 10 mL for determination. This was the bound phenolics of RP and WP by acid hydrolysis (A1 and A2).

#### 2.3.3. Bound Phenolics by Base Hydrolysis

The dried residue (0.5 g) generated after free phenolics extraction was hydrolyzed with 15 mL of NaOH (3 M) solution containing 10 mM EDTA-2 Na and 1% ascorbic acid filled with nitrogen gas. The mixture was incubated in a shaking water bath for 4 h at 30 °C, then adjusted the pH to 2 using HCl (6 M), centrifuged at 10,000× *g* for 15 min. The supernatant was extracted with 20 mL of EA four times. All of the supernatants were concentrated with a rotary evaporator at 45 °C avoiding light, and then added 70% methanol to constant volume of 10 mL for determination. This was the bound phenolics of RP and WP by base hydrolysis (B1 and B2).

#### 2.3.4. Bound Phenolics by Composite Enzymes Hydrolysis

The dried residue (0.5 g) generated after free phenolics extraction was mixed with 0.03 g of composite enzymes (cellulase:hemicellulase:pectinase = 1:1:1), adding 10 mL of H_2_O (adjusted to pH = 5.0 using citric acid) to the mixture, which was incubated in a 50 °C shaking water bath for 2 h. After the enzymatic hydrolysis, the mixtures were kept at oven for 10 min at 80 °C to inactivate the enzymes, then ultrasonic extracted for 30 min at 50 °C with 320 W of ultrasonic power [13]. Before centrifuged at 12,000× *g* for 10 min, a water/ice bath was used to cool the mixtures to 25 °C. The supernatant was extracted with 20 mL of EA four times. All of the supernatants were concentrated with a rotary evaporator at 45 °C avoiding light, and then added 70% methanol to constant volume of 10 mL for determination. This was the bound phenolics of RP and WP by composite enzymes hydrolysis (E1 and E2).

### 2.4. Analyses of Total Phenolic and Total Flavonoid Contents

The concentration of total phenolic compounds was determined using Folin–Ciocalteu reagent according to Singh et al. [22] with slight modification. Briefly, 150 µL of extracts was added to 3 mL distilled water, and oxidized with 500 µL of Folin-Ciocalteu reagent for 8 min at room temperature. Then, the reaction was neutralized with 700 µL of 15% sodium carbonate added with mixing. The solution was incubated at 30 °C for 60 min before taking the absorbance at 765 nm. All tests were performed in triplicates. Gallic acid (10–100 μg/mL) was used as the standard (*R*^2^ = 0.999). The results were expressed as mg GAE (gallic acid equivalents)/g DW (dry weight) of the sample extract.

The concentration of total flavonoids compounds was determined using aluminium chloride procedure referred to Pascoa et al. [23] with slight modification. 500 µL of extracts was added to 2 mL methanol, mixed with 150 µL of 5% NaNO_2_ solution (*w*/*v*). After 6 min, 150 µL of 10% AlCl_3_ solution (*w*/*v*) was added. 2 mL of NaOH (1M) was added to terminate the reaction after 6 min. The solution was incubated at room temperature for 20 min before taking the absorbance at 510 nm. All tests were performed in triplicates. Rutin (10–100 μg/mL) was used as the standard (*R*^2^ = 0.999). The results were expressed as mg RE (Rutin equivalents)/g DW (dry weight) of the sample extract.

### 2.5. UPLC-TOF-MS Analysis

Q-Exactive Obitrap MS (Thermofisher Scientific, Shanghai, China) coupled to an electrospray ionisation (ESI) source was used to elute phenolic compounds. Separation of polyphenols was carried out on an Agilent ZORBAX Eclipse Plus C18 (2.1 × 100 mm, 1.8 μm). The mobile phases A and B were 0.2% formic acid in Milli-Q grade water and 100% acetonitrile, respectively. The gradient was as follows: 0–10 min 10–30% B, 10–15 min 30% to 90% B, 15–16 min 90% to 10% B, 16–20 min 10% B. The flow rate was set at 0.3 mL/min and injection volume was 3 μL. Capillary voltage was maintained at 3200 V. Nitrogen was used as sheath gas flow 30 arb and aux gas flow10 arb. Mass spectra (MS) spectra were recorded in negative ion mode, in the range of 105–1100 *m*/*z*.

Identification of phenolics in the extracts was achieved by comparing their spectra and retention times with those of externally injected standards (Table 1): rutin > 99%; gallic acid, chlorogenic acid, cryptochlorogenic acid, kaempferol, caffeic acid, syringic acid, epicatechin, *p-*coumaric acid, sinapic acid, *p*-hydroxycinnamic acid, nicotiflorin, isoferulic acid, ferulic acid, grosvenorine, isorhamnetin, baicalein > 98%; astragalin, 1,3-dicaffeoylquinic acid, gentiopicrin and diosmin > 97%; isoquercitrin, quercitrin, 4-methoxysalicylic acid > 95%. The precise mass of the parent ion (M−H) and typical MS fragmentation pattern were used to identify compounds which standards were not available according to references. The concentration of each phenolic compound in the extracts was measured with a standard. For quantification, external calibration curves were prepared for each standard.

### 2.6. Antioxidant Activity

The DPPH radical scavenging assay followed the method reported by Arnab et al. [24] with slight modification. A 50 μL volume of sample was mixed with 400 μL DPPH methanolic solution (100 μM) and allowed to stand at room temperature for 30 min under light protection. Each solution (200 μL) was added into a 96-well plate and absorbances were measured at 517 nm using a microplate reader. Methanol was used as a blank control. Trolox (10–150 μg/mL) was used as the standard (*R*^2^ = 0.991). The results were expressed as μmol TE (Trolox equivalents)/g DW (dry weight) of the sample extract.

The ABTS cation radical scavenging activity followed the method reported by van der Werf et al. [25]. Briefly, 10 mL ABTS solution (7.0 mM) was mixed with 176 μL potassium persulfate solution (140 mM). After incubated at room temperature for 12 h in darkness, the stock solution was diluted with PBS (0.05 M K_2_HPO_4_, 0.05 M KH_2_PO_4_, pH 6.8) until the absorbance was 0.7 ± 0.02. A 200 μL volume of sample was mixed with 4 mL diluted ABTS^+^ stock solution and allowed to stand at room temperature for 6 min under light protection. Each solution (200 μL) was added into a 96-well plate and absorbances were measured at 734 nm using a microplate reader. Trolox (10–100 μg/mL) was used as the standard (*R*^2^ = 0.993). The results were expressed as μmol TE (Trolox equivalents)/g DW (dry weight) of the sample extract.

The ferric reducing antioxidant power (FRAP) assay followed method reported by Chen et al. [26]. Briefly, the FRAP reagent was prepared by mixing acetate buffer (300 mM, pH 3.6), a solution of 10 mM TPTZ in 40 mM HCl and 20 mM FeCl_3_ at 10:1:1 (*v*/*v*/*v*). Reagents were manufactured freshly and warmed to 37 °C in a water bath. A 30 μL volume of sample was mixed with 900 μL FRAP solutions and allowed to stand at room temperature for 30 min under light protection. Each solution (200 μL) was added into a 96-well plate and absorbances were measured at 593 nm using a microplate reader. FeSO_4_·7H_2_O (0–800 μmol/mL) was used as the standard (*R*^2^ = 0.999). The results were expressed as μmol Fe(II)SE (ferrous sulfate equivalents)/g DW (dry weight) of the sample extract.

### 2.7. Statistical Analysis

All experiments were carried out in triplicate and the data were expressed as mean ± standard deviation. SPSS (version 26.0.) was used to perform statistical analyses. ANOVA test (Tuckey’s and Bonferroni) were applied to compare means. Differences were considered significant at the *p* < 0.05 level.

## 3. Results and Discussion

### 3.1. Free and Bound Polyphenols Extracted by Different Methods

**Figure 2:**
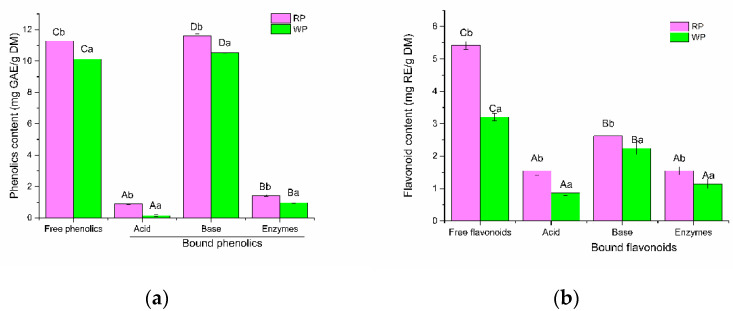
(**a**) The total phenolic contents released from RP or WP after different treatments; (**b**) The flavonoid contents released from RP or WP after different treatments. A–D: statistically significant differences among different extraction methods for RP or WP; a, b: statistically significant differences between RP and WP samples at different extraction methods. RP, red-pulp pitahaya peel; WP, white-pulp pitahaya peel. GAE, Gallic acid equivalents. RE, Rutin equivalents.

Phenolic compounds can be divided into free and bound forms, depending on whether they occur in the free form or are covalently bound to other molecules [27]. Free phenolic compounds are easily extracted by organic solvents, while a large amount of bound phenolics are difficult to extract [13]. In the present study, the contents and compositions of free phenolics by conventional methods (80% methanol extraction) and bound phenolics released by various hydrolysis methods (acid, base, and composite enzymes) in RP and WP were analyzed, respectively. As shown in Figure 2a, free phenolic contents of RP and WP was 11.3 mg GAE/g DW and 10.1 mg GAE/g DW, respectively. The content of free flavonoids in RP (5.4 mg RE/g DW) was significantly (*p* < 0.05) higher than that in WP (3.3 mg RE/g DW) (Figure 2b). The results indicated that the species of pitahaya fruit had influence on the contents of free phenolics. Free flavonoid contents in pitahaya fruit peel were slight lower than that reported by Kim, Choi, Moon, Kim, Mosaddik and Cho [17]. It may be due to the differences in species and cultivated regions of pitahaya fruit samples.

Bound phenolic content in the basic extracts was 11.6 mg GAE/g DW for RP and 10.5 mg GAE/g DW for WP. The contribution of bound phenolics to total phenolics (free and bound) in RP and WP was 50.72% and 50.95%, respectively. The data obtained indicated that the polyphenols content of pitahaya fruit peel has been underestimated in the literature. As shown in Figure 2, hydrolysis methods exhibited significant impacts on the release efficiency of bound phenolics. The amounts of bound phenolics released by acidic hydrolysis (0.9 mg GAE/g DW and 0.1 mg GAE/g DW for RP and WP, respectively) were robustly lower than that of basic hydrolysis. The bound flavonoid amounts released by acidic hydrolysis were 1.6 mg RE/g DW for RP and 0.9 mg RE/g DW for WP. Similarly, the amounts of bound phenolics released by composite enzyme hydrolysis were extremely low (1.4 mg GAE/g DW for RP and 1.0 mg GAE/g DW for WP). The bound flavonoid amounts released by composite enzyme hydrolysis were 1.6 mg RE/g DW and 1.2 mg RE/g DW, respectively. Importantly, base hydrolysis yielded the highest bound phenolic contents, which was 13.04-fold and 8.18-fold for RP and 75.07-fold and 10.94-fold for WP compared with acid and enzymatic hydrolysis, respectively. It also yielded the highest bound flavonoid contents (2.6 mg RE/g DW for RP and 2.2 mg RE/g DW for WP), which was 1.69-fold and 1.69-fold for RP and 2.57-fold and 1.95-fold for WP compared with acid and enzymatic hydrolysis, respectively. Moreover, the contents of phenolics and flavonoids in RP were higher than those of WP varieties by the correspondingly same hydrolysis methods (*p* < 0.05).

The results suggested that base hydrolysis method was more effective than acid and composite enzymes treatment for releasing the bound phenolics in RP and WP, which was consistent with our previous report [21]. They found that alkaline hydrolysis more efficiently liberated bound phenolic compounds in apple pomace than acid hydrolysis. Kim et al. [28] also reported that alkaline hydrolysis released more phenolics from wheat bran than acid hydrolysis. However, the results were different from another previous report [13], in which acidic hydrolysis exhibited more effective ability than base hydrolysis for liberating the bound phenolic compounds in *Rubus idaeus* L. leaves and seeds. It may ascribe to the difference in bond types by which bound phenolics linking to plants matrices in different food as well as the different food matrices. Base hydrolysis is efficient to break the ether and ester bonds linking phenolic compounds to the cell wall, which widely distributed in fruit peel [8]. However, acid hydrolysis mainly breaks glycosidic bonds. Moreover, Verma et al. [29] reported that acid hydrolysis at elevated temperature resulted in the loss of some phenolics. This may result in more efficiency of base hydrolysis than acidic hydrolysis to release bound phenolics from RP and WP.

### 3.2. Identification of Phenolic Compositions in RP and WP

Individual phenolic components liberated from RP and WP were identified and quantified by UPLC-TOF-MS method. The identified compositions of phenolics are shown in Table 2. Up to 37 individual phenolic compounds were detected and tentatively categorized based on their retention time, accurate mass parent ion peaks and secondary fragment ions including 5 hydroxybenzoic acids and derivatives, 11 hydroxycinnamic acids and derivatives, 19 flavonoids and two other substances. In total, 14 phenolic compounds were confirmed and validated with the standards including quercetin, kaempferol, gallic acid, chlorogenic acid, cryptochlorogenic acid, caffeic acid, syringic acid, epicatechin, *p*-coumaric acid, rutin, isoquercitrin, ferulic acid, nicotiflorin, and astragalin.

According to the peak with parent ion, *m*/*z* 169.00 (M−H) corresponded to fragment ions at *m*/*z* 125.05 and 78.95; compound **1** was positively identified as gallic acid. Compound **2** was very likely to be 4-methoxysalicylic acid with parent ion 167.03 (M−H), and thus creating fragment ions at *m*/*z* 123.04 [(M−H)−CO_2_]^−^, due to loss of a CO_2_. Compound **3** and compound **6** were characterized by the parent ion *m*/*z* 353.09 (M−H) and their secondary fragment ions *m*/*z* at 173.05 and 135.04. Compared with the retention time of standards, compound **3** and compound **6** were positively identified as chlorogenic acid and cryptochlorogenic acid, respectively. The parent ion of compound **4** was *m*/*z* 197.04 and fragments iron at *m*/*z* 153.05, and was tentatively confirmed as syringic acid [30]. Compound **5** was tentatively identified as quinic acid with parent ion *m*/*z* 191.06 (M−H), and thus creating fragment ions at *m*/*z* 127.04 [(M−H)−2CH_2_OH]^−^. Compound **7** was very likely to be *p*-hydroxycinnamic acid with parent ion *m*/*z* 163.04 (M−H), and thus creating fragment ions at *m*/*z* 119.05 [(M−H)−CO_2_]^−^, due to loss of a CO_2_. Compound **8** was tentatively identified as gentipicrin with parent ion *m*/*z* 355.10 (M−H), and its creating ions fragments *m*/*z* at 149.06 [(M−H)−glc−CO_2_]^−^, due to the loss of a hexosyl residue and CO_2_. Compound **9** was identified as esculetin with parent ion *m*/*z* 177.02 (M−H), and thus creating fragment ions at *m*/*z* 149.02 [(M−H)−CO]^−^. As the loss of a hexosyl residue, compound **10** ([M−H]^−^ at *m*/*z* 327.11) created fragment ions at *m*/*z* 165.05 and be identified as androsin. By comparing the retention time of standard compounds, compound **11**, **14**, **16**, **18**, **20**, **22**, **26**, **28**, **31** and **35** were identified as caffeic acid, epicatechin, rutin, *p*-coumaric acid, isoquercetin, ferulic acid, astragalin, isoferulic acid, quercetin and kaempferol, respectively. Compound **12** was identified as methyl vanillate with parent ion *m*/*z* 181.05 (M−H), and its creating ions fragments *m*/*z* at 166.03 [(M + H)−H_2_O]^+^. Compound **13** was identified as 7,8-dihydroxycoumarin with parent ion *m*/*z* 177.02 (M−H), and thus creating fragment ions at *m*/*z* 149.02 [(M−H)−CO]^+^. Compound 15 was very likey to be grosvenorine with parent ion *m*/*z* 739.21 (M−H), and one fragment ion at *m*/*z* 285.04. Compound **17** was very likely to be typhaneoside by its parent ion *m*/*z* 769.22 and two fragment ions at *m*/*z* 314.04 and 151.00. Compound **19** was identified as lonicerin with parent ion *m*/*z* 593.15 (M−H), and thus creating fragment ions at *m*/*z* 447.09 [(M−H)−2C_3_H_7_OCH_2_]^−^ and *m*/*z* 285.04 [(M−H)−2C_3_H_7_OCH_2_−glc]^−^ Compound **21** was tentatively identified as sinapic acid according to the ion of *m*/*z* 223.06 and its secondary fragment ions *m*/*z* at 193.01 and 149.02 [31]. Compound **23** and **24** gave *m*/*z* 593.15, which corresponded to formula C_27_H_30_O_15_ and had kaempferol characteristic fragment ions, were identified as kaempferol-3-o-rutinoside (nicotiflorin) and kaempferol-3-glucorhamnoside, respectively, based on the similar fragmentation pattern reported by Zulkifli, Abd Gani, Zaidan and Halmi [31] and Correa-Betanzo et al. [32]. Compound **25** was tentatively identified as isorhamnetin-3-o-neohesperidine according to the ion of *m*/*z* 623.16 and secondary fragment ions *m*/*z* at 314.04 and 271.02. Compound **32** was identified as methyl 4-hydroxycinnamate with parent ion *m*/*z* 177.06 (M−H), and thus creating fragment ions at *m*/*z* 145.03 [(M−H) −CH_2_OH]^−^. Compound **27** was very likely to be 1,3−dicaffeoylquinic acid with parent ion *m*/*z* 515.12 (M−H), and thus creating fragment ions at *m*/*z* 353.09 [(M−H)−glc]^−^ and *m*/*z* 191.06 [(M−H)−2glc]^−^. Compound **29** was tentatively to be diosmin with parent ion *m*/*z* 607.17 (M−H), and thus creating one fragment ion at *m*/*z* 299.06 [(M−H)−2C_3_H_7_OCH_2_−glc]^−^. Compound **30** was identified as abscisic acid with parent ion *m*/*z* 263.13 (M−H), and thus creating fragment ions at *m*/*z* 219.14 [(M−H)−CO_2_]^−^ and *m*/*z* 163.08 [(M−H)−C_4_H_9_COCH_2_]^−^. Compound **33** was identified as calycosin with parent ion *m*/*z* 283.06 (M−H), and thus creating fragment ions at *m*/*z* 268.04 [(M + H)−H_2_O]^+^. Compound **35** was tentatively identified as isorhamnetin according to the ion of *m*/*z* 313.05 and its MS/MS fragment ions at 300.03 and 151.00 [33]. Compound **36** was identified as baicalein with parent ion *m*/*z* 269.05 (M−H), and its creating ions fragments *m*/*z* at 251.03 [(M−H)−H_2_O]^−^, due to the loss of a H_2_O. As the loss of a CH_3_, compound **37** ([M−H]^−^ at *m*/*z* 299.06) created fragment ions at *m*/*z* 284.03 and be identified as tectorigenin.

### 3.3. Quantity of Predominant Individual Phenolic Compounds in Various Extracts

Table 3 showed contents of predominant individual phenolic compounds released from RP and WP following different hydrolysis methods. The absolute amounts of the individual phenolic compounds detected were between 0.05–131.67 mg/kg DW. Among all the free and bound phenolics, the major phenolic compounds in RP identified were chlorogenic acid (19.14–19.45 mg/kg DW), ferulic acid (10.72–25.04), *p*-coumaric acid (8.62–16.29) and caffeic acid (5.44–57.03), whereas chlorogenic acid (132.71–133.88), caffeic acid (12.96–33.16), rutin (11.58–11.93), isoquercetin (10.73–11.28) and ferulic acid (7.91–21.52) were the main compounds in WP. The major free phenolic compounds in the 80% methanol extract for RP (F1) identified were chlorogenic acid (17.75 mg/kg DW), quercetin (7.18), ferulic acid (6.68), *p*-coumaric acid (4.96) and nicotiflorin (4.18), whereas the major free phenolics for WP (F2) were chlorogenic acid (131.67), rutin (11.03), isoquercetin (10.30), caffeic acid (7.73), and ferulic acid (5.27). For bound phenolics released by acid, base and composite enzymes extraction, the major individual phenolics were all the same substances-caffeic acid, ferulic acid and *p*-coumaric acid. Extraction by base hydrolysis of RP (B1) and WP (B2) released most caffeic acid (54.18 for B1, 25.43 for B2), ferulic acid (18.36 for B1, 16.25 for B2) and *p*-coumaric acid (11.33 for B1, 6.63 for B2). While for acid and composite enzymes hydrolysis extracts, the major phenolics were ferulic acid (9.88 for A1, 5.27 for A2, 4.04 for E1, 2.64 for E2), *p*-coumaric acid (3.66 for A1, 3.48 for A2, 5.56 for E1, 3.09 for E2), and caffeic acid (2.59 for A1, 5.23 for A2, 3.4 for E1, 5.35 for E2). Different hydrolysis methods have a great influence on the content of individual phenolic compounds extracted from pitahaya peel. As present in Table 3, the quantitative results showed that many individual phenolics were increased after base hydrolysis, including caffeic acid (19-fold for RP, 3.29-fold for WP), *p-*coumaric acid (2.28-fold for RP, 1.91-fold for WP), and ferulic acid (2.75-fold for RP, 3.08-fold for WP). Compared with the other two methods, base hydrolysis extraction exhibited the biggest potential to release bound individual phenolic compounds. In addition, the difference in kinds and content of individual phenolics between two species was ascribed to the species heredity.

### 3.4. Antioxidant Activity of Phenolics in RP and WP

A previous research reported that free phenolic compounds in the pulp of two species of pitahaya fruits exhibited a high antioxidant activity [20]. However, the antioxidant activity of bound phenolics in pitahaya peel remained unknown. In the present work, the ABTS^+^, DPPH radical scavenging activity and ferric reducing antioxidant activity (FRAP) were accordingly measured to estimate the antioxidant activity of bound phenolics released from RP and WP following different hydrolysis. As shown in Table 4, free phenolics extracts showed high ABTS^+^, DPPH and FRAP values (13.03 μmol TE/g DW, 6.82 μmol TE/g DW and 102.69 μmol Fe(II)SE/g DW for RP, 13.43 μmol TE/g DW, 7.01 μmol TE/g DW and 107.99 μmol Fe(II)SE/g DW for WP). In bound phenolics extracts of RP, it was observed that the base hydrolysis extracts retained the highest ABTS^+^ and DPPH values (33.62 μmol TE/g DW for ABTS^+^, 31.34 μmol TE/g DW for DPPH and 237.25 μmol Fe(II)SE/g DW for FRAP) (*p* < 0.01), while the extracts obtained by composite enzymes hydrolysis and the acid hydrolysis both showed low ABTS^+^ and DPPH values. Concerning WP, the ABTS^+^, DPPH and FRAP values of bound phenolics from base hydrolysis were obviously higher than those from the other two hydrolysis methods (*p* < 0.01). Previous studies showed that the antioxidant activity of polyphenols-rich extracts from plant was closely related to the presence of phenolic compounds [34]. After extracted by 80% methanol, base hydrolysis released a large number of phenolic compounds. Nevertheless, acid and composite enzymes hydrolysis displayed a low efficiency in releasing the bound phenolics in RP and WP, which may result in the low antioxidant activities. Additionally, caffeic acid and ferulic acid were found to possess the stronger antioxidant activity compared with several other individual phenolic compounds [29]. The high amounts of caffeic acid and ferulic acid was released by base hydrolysis, which may contribute to the stronger antioxidant activity of extracts from base hydrolysis compared to the two other hydrolysis methods. Considering the comparatively higher antioxidant activities and lower content of bound phenolics by base hydrolysis compared with free phenolics, our results indicated that the comparatively high concentration of caffeic acid, *p*-coumaric acid and syringic acid in bound phenolics may play an important role in the antioxidant activities performance of RP and WP. Consequently, base hydrolysis method was the most efficient extraction to liberate the antioxidative phenolic components from RP and WP.

### 3.5. Correlation between Phenolic Compounds and Antioxidant Activity

Pearson’s correlation coefficient among DPPH, ABTS^+^, FRAP, TPC, TFC and predominant individual phenolic of pitahaya peel was presented in Table 5. There were significant (*p* < 0.01) positive correlations between the TPC and the antioxidant activities (r = 0.717, 0.803, 0.872 in RP, r = 0.764, 0.784, 0.871 in WP for DPPH, ABTS^+^ and FRAP, respectively), implying that the antioxidant capacity of pitahaya fruit peel mainly originated from its phenolic substances. The positive relationship between total phenolics content and antioxidant activity was also reported previously [35]. A direct correlation was found between the phenolic contents and antioxidant effects in the free phenolics (by methanol extraction) of pulp and peel of white and red pitahayas, collected from Jeju Island, Korea [17]. However, no significant correlation emerged between TFC and the antioxidant activities in RP and WP. This was probably due to the comparatively low flavonoid contents compared with its phenolic acid contents in RP and WP. Considering the comparatively high phenolic acids content in bound form, our results implied that phenolic acids liberated from RP and WP played important roles in the antioxidant activity performance.

Phenolic compounds in plant were already reported to be effective scavengers of free radicals. However, there is no information about the contribution of individual phenolics to their overall antioxidant capacity in pitahaya peel. To further investigate the contribution of predominant individual phenolic compound to the antioxidant capacity of phenolic extracts of RP and WP, the correlation analysis was established (Table 5). The main phenolics contributors to antioxidant capacities in WP seem to be caffeic acid > *p*-coumaric acid > syringic acid. For RP, it seems to be interesting that the main phenolics contributors to antioxidant capacities were in the following order: syringic acid > caffeic acid > *p*-coumaric acid > ferulic acid > rutin > isoquercitrin. It can also be noticed that chlorogenic acid, nicotiflorin and quercetin appeared to not contribute to the antioxidant activity for the ABTS, DPPH, and FRAP assays both in RP and WP. Unlike the RP, ferulic acid, rutin and isoquercitrin were not significant contributors for the ABTS, DPPH, and FRAP assays (*p* > 0.05) in WP. Arruda et al. [36] reported that chlorogenic acid showed few contributions to the antioxidant activity in the pulp and peel of araticum fruit. The comparatively high content of chlorogenic acid in free phenolics may result in its low value of antioxidant activities. Previous studies have shown that individual phenolic compounds present in the extracts may exert their antioxidant activity individually as well as synergistically or antagonistically [37]. This may cause individual phenolic compounds exhibited different contributes to the antioxidant activities in RP and WP.

## 4. Conclusions

This is the first report to systematically investigate the compositions and contents of bound phenolics of red-pulp and white-pulp pitahaya peel treated by different hydrolysis methods (acid, base and composite enzymes). The results indicated that base hydrolysis was the highest efficient method for releasing the bound phenolics in RP and WP. Chlorogenic acid, quercetin and ferulic acid were the major free phenolic compounds, whereas caffeic acid, ferulic acid and *p*-coumaric acid were the predominant bound phenolics in RP and WP. In addition, the antioxidant capacity of RP and WP showed a positive correlation with the total phenolics, and with specific individual phenolic compounds such as caffeic acid, ferulic acid, syringic acid and *p*-coumaric acid. These findings showed that hydrolysis methods had substantial effects on the release of bound phenolics in pitahaya peel which can be a promising source of natural antioxidants by effective extraction.

## Figures and Tables

**Table 1:** Calibration curves used for UPLC-MS/MS quantification of polyphenols.

Phenolic Compounds	Calibration Curves	Correlation Coefficients (r^2^)	Linear Ranges (ng/mL)	LOD (ng/mL)	LOQ (ng/mL)	Recovery (%)
astragalin	Y = −4,803,060 + 287,294X	1.0000	21.560–5000.342	5.917	19.723	98.97–101.79
syringic acid	Y = −5,794,220 + 81,951.2X	0.9992	74.706–5019.928	20.742	69.141	96.74–99.92
gallic acid	Y = −10,582,100 + 321,771X	0.9997	198.933–5009.727	49.897	166.323	96.57–100.42
chlorogenic acid	Y = −3,822,170 + 74,204.2X	0.9991	52.036–5022.678	9.427	31.423	97.14–99.97
cryptochlorogenic acid	Y = −4,693,760 + 116,490X	0.9996	40.436–5007.027	5.864	19.547	98.61–102.67
kaempferol	Y = −24,043,800 + 519,101X	0.9996	90.191–5012.449	21.218	70.727	101.21–103.09
caffeic acid	Y = −57,997,300 + 977,935X	1.0000	161.473–4998.604	39.659	132.196	99.57–101.09
ferulic acid	Y = −28,597,200 + 367,571X	0.9999	85.506–5004.645	20.276	67.587	102.31–103.97
epicatechin	Y = −2,607,660 + 417,721X	0.9999	10.692–4992.796	2.969	9.897	99.83–101.96
*p*-coumaric acid	Y = −68,269,600 + 786,892X	0.9998	87.599–5000.646	24.259	80.863	97.13–99.93
rutin	Y = −4,547,420 + 167,759X	0.9998	27.313–5011.457	5.987	19.959	97.97–103.15
isoquercitrin	Y = −5,259,570 + 244,998X	0.9999	21.642–5003.646	5.362	17.873	99.48–100.37
quercitrin	Y = −3,409,400 + 325,845X	0.9997	62.223–5009.821	12.518	41.727	98.31–101.37
nicotiflorin	Y = −2,387,800 + 190,613X	1.0000	12.898–5003.779	3.337	11.123	99.35–100.01
4-methoxysalicylic acid	Y = −632,177 + 55,830X	0.9997	32.875–3979.501	5.810	19.367	94.81–97.56
sinapic acid	Y = 5,735,810 + 107,181X	0.9995	30.237–5991.023	6.095	20.317	94.87–99.37
*p*-hydroxycinnamic acid	Y = 2,185,610 + 80,817.5X	0.9997	27.690–5095.187	6.397	21.323	96.64–102.32
1,3-dicaffeoylquinic acid	Y = −5,672,173 + 57,810X	0.9951	32.902–3979.102	6.356	21.187	97.31–101.59
isoferulic acid	Y = −1,535,870 + 173,911X	1.0000	56.769–4908.780	11.839	39.463	98.86–101.16
gentiopicrin	Y = −1,104,055 + 179,871.4X	0.9997	50.810–3993.299	12.119	40.397	99.17–100.31
grosvenorine	Y = −1,001,090 + 245,481.9X	1.0000	52.007–5001.712	11.852	39.507	95.31–102.16
diosmin	Y = −3,091,250 + 315,432.9X	0.9997	73.502–5019.121	9.737	32.457	96.17~99.01
isorhamnetin	Y = −7,171,780 + 227,089X	0.9995	57.201–3911.709	15.011	50.037	98.97–100.67
baicalein	Y = −3,381,572 + 285,915X	0.9997	23.712–5095.538	6.095	20.317	97.17–102.17

**Table 2:** Identification of the main phenolic compositions released from RP and WP following different extractions.

No.	RT (min)	Compounds	Formula	*m*/*z* [M−H]	*m*/*z* Fragments	Phenolics Fractions
		Hydroxybenzoic acids and derivatives				
**1**	4.73	gallic acid ^a,b^	C_7_H_6_O_5_	169.00	125.05, 78.95	F1, F2, A1, A2, B1, B2, E1
**2**	5.29	4-methoxysalicylic acid ^a,b^	C_8_H_8_O_4_	167.03	123.04	E2
**4**	7.31	syringic acid ^a,b^	C_9_H_10_O_5_	197.04	153.05	F1, F2, A1, A2
**12**	8.67	methyl vanillate ^b^	C_9_H_10_O_4_	181.05	166.03	F2
**21**	10.97	sinapic acid ^a,b^	C_11_H_12_O_5_	223.06	193.01, 149.02	B1
		Hydroxycinnamic acids and derivatives				
**3**	7.30	chlorogenic acid ^a,b^	C_16_H_18_O_9_	353.09	161.02, 191.06	F1, F2, A1, A2, B1, B2, E1, E2
**6**	7.33	cryptochlorogenic acid ^a,b^	C_16_H_18_O_9_	353.09	173.05, 191.06	A1, A2, B1, B2, E2
**7**	7.35	*p*-hydroxycinnamic acid ^a,b^	C_9_H_8_O_3_	163.04	119.05	F1
**9**	8.15	esculetin ^b^	C_9_H_6_O_4_	177.02	149.02	B1
**11**	8.34	caffeic acid ^a,b^	C_9_H_8_O_4_	179.03	135.04, 107.05	F1, F2, A1, A2, B1, B2, E1, E2
**13**	8.88	7,8-dihydroxycoumarin ^b^	C_9_H_6_O_4_	177.02	149.02	F1, B1
**18**	10.28	ρ-coumaric acid ^a,b^	C_9_H_8_O_3_	163.04	119.05	F1, F2, A1, A2, B1, B2, E1, E2
**22**	11.06	ferulic acid ^a,b^	C_10_H_10_O_4_	192.05	134.04, 178.03	F1, F2, A1, A2, B1, B2, E1, E2
**27**	12.65	1,3-dicaffeoylquinic acid ^a,b^	C_25_H_24_O_12_	515.12	353.09, 191.06	F2
**28**	13.74	isoferulic acid ^a,b^	C_10_H_10_O_4_	193.05	134.04, 178.03	B1, B2, E1, E2
**32**	16.48	methyl 4-hydroxycinnamate ^b^	C_10_H_10_O_3_	177.06	145.03	F1, F2, A2, E1, E2
		Flavonoids				
**8**	7.96	gentiopicrin ^a,b^	C_16_H_20_O_9_	355.10	149.06	F1
**10**	8.15	androsin ^b^	C_15_H_20_O_8_	327.11	165.05	F2
**14**	9.37	Epicatechin ^a,b^	C_15_H_14_O_6_	289.10	244.90, 108.90	F1, F2
**15**	9.73	grosvenorine ^a,b^	C_33_H_40_O_19_	739.21	285.04	F1
**16**	9.78	rutin ^a,b^	C_27_H_30_O_16_	609.15	300.03	F1, F2, A1, A2, B1, B2, E1, E2
**17**	9.83	typhaneoside ^b^	C_34_H_42_O_20_	769.22	314.04, 151.00	F1, F2, B1, B2, E2
**19**	10.64	lonicerin ^b^	C_27_H_30_O_15_	593.15	447.09, 285.04	F1, F2
**20**	10.95	isoquercetin ^a,b^	C_21_H_20_O_12_	463.09	300.03, 271.03	F1, F2, A1, A2, B1, B2, E1, E2
**23**	11.49	nicotiflorin ^a,b^	C_27_H_30_O_15_	593.15	285.04	F1, F2, A1, A2, B1, B2, E1, E2
**24**	11.50	kaempferol 3-glucorhamnoside ^b^	C_27_H_30_O_15_	593.15	284.03, 327.05	E2
**25**	11.72	isorhamnetin-3-O-neohesperidine ^b^	C_28_H_32_O_16_	623.16	314.04, 271.02	F1, F2, B1, B2, E1, E2
**26**	12.12	astragalin ^a,b^	C_21_H_20_O_11_	447.09	284.03, 227.03	F1, F2, B2, E1, E2
**29**	13.97	Diosmin ^a,b^	C_28_H_32_O_15_	607.17	299.06	F2
**31**	15.95	quercetin ^a,b^	C_15_H_10_O_7_	301.04	151,179	F1, F2, A1, A2, B1, E1, E2
**33**	16.58	calycosin ^b^	C_16_H_12_O_5_	283.06	268.04	F1
**34**	17.55	kaempferol ^a,b^	C_15_H_10_O_6_	285.04	151.00	F1, F2, A1, A2, B1, B2, E1, E2
**35**	17.76	isorhamnetinab	C_16_H_12_O_7_	315.05	300.03, 151.00	F2, A1, A2, E1, E2
**36**	17.93	baicalein ^a,b^	C_15_H_10_O_5_	269.05	251.03	F2
**37**	18.12	tectorigenin ^b^	C_16_H_12_O_6_	299.06	284.03	F2, A1
		Others				
**5**	7.32	quinic acid ^b^	C_7_H_12_O_6_	191.06	127.04	F2
**30**	15.12	abscisic acid ^b^	C_15_H_20_O_4_	263.13	163.08, 219.14	B1

^a^ compared with reference standards, ^b^ compared with references. F1: Free phenolics of RP by methanol extraction; F2: Free phenolics of WP by methanol extraction; A1: Bound phenolics of RP by acid hydrolysis; A2: Bound phenolics of WP by acid hydrolysis; B1: Bound phenolics of RP by base hydrolysis; B2: Bound phenolics of WP by base hydrolysis; E1: Bound phenolics of RP by composite enzymes hydrolysis; E2: Bound phenolics of WP by composite enzymes hydrolysis.

**Table 3:** Content of predominant individual phenolic compounds released from RP and WP following different extractions.

Class	Sub-Class	Analytes	Contents (mg/kg DW)			
			Red-pulp pitahaya peel			
			F1	A1 (%)	B1 (%)	E1 (%)
Phenolic acids	Hydroxybenzoic acids	gallic acid	1.45 ± 0.02	1.50 ± 0.76 (50.85)	1.42 ± 0.72 (49.48)	0.95 ± 0.51 (39.58)
		4-methoxysalicylic acid	0.53 ± 0.01	0.35 ± 0.00 (39.77)	0.55 ± 0.01 (50.93)	0.23 ± 0.01 (30.26)
		syringic acid	2.43 ± 0.13	1.77 ± 0.04 (42.14)	ND	ND
		sinapic acid	1.15 ± 0.01	1.05 ± 0.31 (47.73)	1.25 ± 0.72 (52.08)	0.35 ± 0.51 (23.33)
	Hydroxycinnamic acids	chlorogenic acid	17.75 ± 1.09	1.54 ± 0.06 (7.98)	1.70 ± 0.95 (8.74)	1.39 ± 0.05 (7.26)
		cryptochlorogenic acid	ND	0.87 ± 0.00 (100.00)	0.85 ± 0.03 (100.00)	ND
		*p*-hydroxycinnamic acid	1.25 ± 0.05	0.85 ± 0.01 (40.48)	1.45 ± 0.07 (53.70)	0.37 ± 0.02 (14.12)
		caffeic acid	2.85 ± 0.25	2.59 ± 0.62 (47.61)	54.18 ± 3.06 (95.00)	3.40 ± 0.41 (54.40)
		*p*-coumaric acid	4.96 ± 0.25	3.66 ± 0.21 (42.46)	11.33 ± 0.90 (69.55)	5.56 ± 0.39 (52.85)
		ferulic acid	6.68 ± 1.27	9.88 ± 1.88 (59.66)	18.36 ± 3.37 (73.32)	4.04 ± 0.73 (37.69)
		1,3-dicaffeoylquinic acid	1.58 ± 0.13	1.57 ± 0.12 (49.84)	1.65 ± 0.07 (51.08)	0.59 ± 0.01 (27.19)
		isoferulic acid	1.65 ± 0.03	1.00 ± 0.11 (37.74)	1.55 ± 0.37 (48.44)	ND
Flavonoids		epicatechin	2.00 ± 0.89	ND	ND	ND
		kaempferol	1.37 ± 0.07	1.32 ± 0.12 (49.07)	1.10 ± 0.21 (44.53)	0.97 ± 0.01 (41.45)
		gentiopicrin	1.07 ± 0.03	1.01 ± 0.10 (48.56)	0.70 ± 0.01 (39.55)	0.57 ± 0.02 (34.76)
		grosvenorine	0.17 ± 0.00	0.31 ± 0.02 (64.58)	0.19 ± 0.02 (52.78)	0.17 ± 0.01 (50)
		diosmin	1.05 ± 0.05	0.59 ± 0.01 (35.98)	0.78 ± 0.01 (42.62)	0.65 ± 0.03 (38.24)
		isorhamnetin	1.09 ± 0.05	ND	0.92 ± 0.03 (45.77)	ND
		baicalein	0.57 ± 0.02	0.32 ± 0.03 (35.96)	0.51 ± 0.03 (47.22)	0.37 ± 0.02 (39.36)
		astragalin	1.19 ± 0.06	ND	0.42 ± 0.01 (26.09)	0.45 ± 0.03 (27.44)
		nicotiflorin	4.18 ± 0.19	0.25 ± 0.00 (5.64)	1.15 ± 0.11 (21.58)	0.64 ± 0.11 (13.28)
		quercetin	7.18 ± 0.12	0.32 ± 0.07(4.27)	0.55 ± 0.02 (7.12)	0.61 ± 0.02 (7.83)
		rutin	1.76 ± 0.00	0.54 ± 0.00(23.48)	1.48 ± 0.11 (45.68)	0.61 ± 0.04 (25.74)
		isoquercitrin	3.04 ± 0.14	0.43 ± 0.00(12.39)	2.73 ± 0.30 (47.31)	0.60 ± 0.04 (16.48)
			White-pulp pitahaya peel			
			F2	A2 (%)	B2 (%)	E2 (%)
Phenolic acids	Hydroxybenzoic acids	gallic acid	1.29 ± 0.09	2.10 ± 2.06 (61.95)	0.74 ± 0.07 (36.45)	ND
		4-methoxysalicylic acid	0.43 ± 0.00	0.15 ± 0.00 (25.86)	0.53 ± 0.011 (55.21)	0.13 ± 0.01 (23.21)
		syringic acid	1.99 ± 0.01	1.69 ± 0.08 (45.92)	ND	ND
		sinapic acid	1.05 ± 0.02	1.01 ± 0.21 (49.03)	1.75 ± 0.32 (62.50)	0.05 ± 0.00 (4.55)
	Hydroxycinnamic acids	chlorogenic acid	131.67 ± 13.50	1.88 ± 0.10 (1.41)	1.04 ± 0.00 (0.78)	2.21 ± 0.19 (1.65)
		cryptochlorogenic acid	ND	0.95 ± 0.15 (100.00)	0.81 ± 0.00 (100.00)	1.06 ± 0.37 (100.00)
		*p*-hydroxycinnamic acid	1.02 ± 0.05	0.75 ± 0.01 (42.37)	1.35 ± 0.05 (56.96)	ND
		*p*-coumaric acid	3.48 ± 0.21	2.34 ± 0.12 (40.21)	6.63 ± 1.92 (65.58)	3.09 ± 0.21 (47.03)
		caffeic acid	7.73 ± 1.39	5.23 ± 1.34 (40.35)	25.43 ± 9.01 (76.69)	5.35 ± 0.71 (40.90)
		ferulic acid	5.27 ± 1.13	5.99 ± 0.92 (53.20)	16.25 ± 1.77 (75.51)	2.64 ± 0.27 (33.38)
		1,3-dicaffeoylquinic acid	1.08 ± 0.03	1.17 ± 0.10 (52.00)	1.55 ± 0.05 (58.94)	0.30 ± 0.00 (21.74)
		isoferulic acid	1.15 ± 0.02	1.01 ± 0.10 (46.76)	1.35 ± 0.30 (54.00)	0.05 ± 0.00 (4.17)
Flavonoids		epicatechin	1.84 ± 0.68	ND	ND	ND
		kaempferol	1.00 ± 0.03	1.49 ± 0.18 (59.84)	1.49 ± 0.18 (59.84)	0.94 ± 0.00 (48.45)
		gentiopicrin	1.01 ± 0.02	0.91 ± 0.03 (47.40)	1.70 ± 0.11 (62.73)	0.37 ± 0.01 (26.81)
		grosvenorine	0.15 ± 0.00	0.11 ± 0.00 (42.31)	0.17 ± 0.01 (53.13)	ND
		diosmin	1.00 ± 0.05	0.39 ± 0.01 (28.06)	1.58 ± 0.05 (61.24)	0.15 ± 0.01 (13.04)
		isorhamnetin	1.09 ± 0.09	ND	0.52 ± 0.03 (32.30)	ND
		baicalein	0.37 ± 0.01	0.22 ± 0.01 (37.29)	0.39 ± 0.05 (51.32)	0.30 ± 0.00 (44.77)
		astragalin	0.63 ± 0.04	ND	ND	0.38 ± 0.01 (37.63)
		nicotiflorin	2.58 ± 0.11	0.25 ± 0.00 (8.83)	0.39 ± 0.06 (13.13)	0.54 ± 0.05 (17.31)
		quercetin	2.26 ± 0.53	1.34 ± 0.24 (37.22)	ND	0.30 ± 0.02 (11.72)
		rutin	11.03 ± 0.50	0.55 ± 0.00 (4.75)	0.79 ± 0.11 (6.35)	0.90 ± 0.04 (7.54)
		isoquercitrin	10.30 ± 1.01	0.43 ± 0.00 (4.01)	0.98 ± 0.30 (8.69)	0.86 ± 0.06 (7.71)

Values are expressed as the mean ± standard deviation. ND: non detected. The number in brackets means the percentage of the total individual phenolic compounds. F1: Free phenolics of RP by methanol extraction; F2: Free phenolics of WP by methanol extraction; A1: Bound phenolics of RP by acid hydrolysis; A2: Bound phenolics of WP by acid hydrolysis; B1: Bound phenolics of RP by base hydrolysis; B2: Bound phenolics of WP by base hydrolysis; E1: Bound phenolics of RP by composite enzymes hydrolysis; E2: Bound phenolics of WP by composite enzymes hydrolysis.

**Table 4:** The ABTS^+^, DPPH radical scavenging activity and ferric reducing antioxidant activity of phenolics released from RP and WP following different extractions.

Stage	Extraction	Red-Pulp Pitahaya Peel	White-Pulp Pitahaya Peel
ABTS^+^ radical scavenging activity (μmol TE/g DW)	Methanol	13.03 ± 0.09 Ba	13.43 ± 0.03 Ba
	Acid	4.56 ± 0.27 Aa	5.03 ± 0.11 Aa
	Base	33.62 ± 2.16 Ca	38.42 ± 1.42 Cb
	Composite enzymes	4.24 ± 0.33 Aa	4.15 ± 0.18 Aa
DPPH radical scavenging activity (μmol TE/g DW)	Methanol	6.82 ± 0.02 Ba	7.01 ± 0.20 Ba
	Acid	2.02 ± 0.20 Aa	1.84 ± 0.16 Aa
	Base	31.34 ± 3.72 Cb	25.19 ± 2.01 Ca
	Composite enzymes	1.28 ± 0.14 Aa	1.08 ± 0.15 Aa
Ferric reducing/antioxidant power (μmol Fe(II)SE /g DW)	Methanol	102.69 ± 3.27 Ca	107.99 ± 1.72 Ca
	Acid	4.23 ± 0.39 Aa	5.62 ± 0.63 Aa
	Base	237.25 ± 3.57 Da	254.2 ± 5.07 Db
	Composite enzymes	13.68 ± 0.47 Ba	12.38 ± 0.32 Ba

Values are expressed as the mean ± SD. Different uppercase letters (A–D) in the same column mean statistically significant differences among different treatment methods for RP or WP (*p* < 0.05). Different lowercase letters (a, b) in the same row mean statistically significant differences between RP and WP samples from different treatment methods (*p* < 0.05).

**Table 5:** Pearson’s correlation coefficient analysis between the antioxidant activity and phenolics of RP and WP from different treatment methods.

	DPPH	ABTS	FRAP
TPC (RP)	0.717 **	0.803 **	0.872 **
TPC (WP)	0.764 **	0.784 **	0.871 **
TFC (RP)	0.110	0.237	0.353
TFC (WP)	0.367	0.435	0.566
Caffeic acid (RP)	0.979 **	0.947 **	0.908 **
Caffeic acid (WP)	0.904 **	0.883 **	0.863 **
Ferulic acid (RP)	0.836 **	0.857 **	0.785 **
Ferulic acid (WP)	0.497	0.548	0.51
Chlorogenic acid (RP)	−0.154	−0.023	0.094
Chlorogenic acid (WP)	−0.111	−0.083	0.067
Syringic acid (RP)	0.968 **	0.966 **	0.960 **
Syringic acid (WP)	0.968 *	0.964 *	0.971 *
Nicotiflorin (RP)	−0.029	0.090	0.214
Nicotiflorin (WP)	−0.176	−0.151	0.002
Rutin (RP)	0.758 *	0.791 *	0.851 *
Rutin (WP)	−0.117	−0.143	0.033
*p*-coumaric acid (RP)	0.947 **	0.929 **	0.904 **
*p*-coumaric acid (WP)	0.874 **	0.862 **	0.859 **
Quercetin (RP)	−0.154	−0.028	0.095
Quercetin (WP)	0.016	0.065	0.203
Isoquercitrin (RP)	0.635 *	0.716 **	0.804 **
Isoquercitrin (WP)	−0.073	−0.045	0.107

TPC, Total phenolic contents; TFC, Total flavonoid contents; * Correlation was significant at the 0.05 level (two-tailed). ** Correlation was significant at the 0.01 level (two-tailed).

## Data Availability

Not applicable.
